# Skeletal muscle myopenia in mice model of bile duct ligation and carbon tetrachloride‐induced liver cirrhosis

**DOI:** 10.14814/phy2.13153

**Published:** 2017-03-31

**Authors:** Michela Giusto, Laura Barberi, Francesca Di Sario, Emanuele Rizzuto, Carmine Nicoletti, Francesca Ascenzi, Anastasia Renzi, Nicola Caporaso, Giuseppe D'Argenio, Eugenio Gaudio, Antonio Musarò, Manuela Merli

**Affiliations:** ^1^ Gastroenterology Department of Clinical MedicineSapienza University of RomeRomeItaly; ^2^ Department of Anatomy, Histology, Forensic Medicine and Orthopedics ‐Unit of Histology and Medical EmbryologySapienza University of RomeLaboratory affiliated to Istituto Pasteur Italia – Fondazione Cenci Bolognetti RomeItaly; ^3^ Department of Mechanical and Aerospace EngineeringSapienza University of RomeRomeItaly; ^4^ Department of Anatomy, Histology, Forensic Medicine and OrthopedicsSapienza University of RomeRomeItaly; ^5^ Department of Clinical and Experimental MedicineFederico II University of NaplesNaplesItaly

**Keywords:** Cirrhosis, molecular pathways, muscle, myopathy

## Abstract

Skeletal muscle myopathy is universal in cirrhotic patients, however, little is known about the main mechanisms involved. The study aims to investigate skeletal muscle morphological, histological, and functional modifications in experimental models of cirrhosis and the principal molecular pathways responsible for skeletal muscle myopathy. Cirrhosis was induced by bile duct ligation (BDL) and carbon tetrachloride (CCl4) administration in mice. Control animals (CTR) underwent bile duct exposure or vehicle administration only. At sacrifice, peripheral muscles were dissected and weighed. Contractile properties of extensor digitorum longus (EDL) were studied in vitro. Muscle samples were used for histological and molecular analysis. Quadriceps muscle histology revealed a significant reduction in cross‐sectional area of muscle and muscle fibers in cirrhotic mice with respect to CTR. Kinetic properties of EDL in both BDL and CCl4 were reduced with respect to CTR; BDL mice also showed a reduction in muscle force and a decrease in the resistance to fatigue. Increase in myostatin expression associated with a decrease in AKT‐mTOR expressions was observed in BDL mice, together with an increase in LC3 protein levels. Upregulation of the proinflammatory citochines TNF‐a and IL6 and an increased expression of NF‐kB and MuRF‐1 were observed in CCl4 mice. In conclusion, skeletal muscle myopenia was present in experimental models of BDL and CCl4‐induced cirrhosis. Moreover, reduction in protein synthesis and activation of protein degradation were the main mechanisms responsible for myopenia in BDL mice, while activation of ubiquitin‐pathway through inflammatory cytokines seems to be the main potential mechanism involved in CCl4 mice.

## Introduction

Muscle wasting is a characteristic feature of liver cirrhosis (O'Brien and Williams 2008) . In spite being a significant clinical complication in these patients, also affecting prognosis, the mechanisms mediating muscle loss are not completely known (Dasarathy 2012). Recently, elevated plasma concentrations of myostatin, a negative regulator of muscle mass, were reported in patients with cirrhosis (García et al. 2010), while contradictory results have been published on the expression of myostatin in muscle biopsy obtained from cirrhotic patients (Merli et al. 2013; Qiu et al. 2013). The difficulty in obtaining muscle biopsy in cirrhotic patients is one of the reasons why knowledge about the pathophysiological mechanism of muscle wasting is still scarce. Therefore, the use of animal models can be fundamental. In the experimental model of liver atrophy secondary to portocaval shunt, hyperammonemia, a frequent alteration in liver cirrhosis, has been proposed as the main trigger able to activate NF‐kB inducing transcription of myostatin (Qiu et al. 2013). In the same model, the expression of ubiquitin‐proteasome pathway was reported to be unaltered, while the expression of autophagy markers was found to be enhanced with respect to control animals (Dasarathy et al. 2004, 2011, 2007; Qiu et al. 2012).

A multifactorial etiology for sarcopenia, secondary to liver cirrhosis, is conceivable (O'Brien and Williams 2008) and the results obtained in the animal models with portocaval shunt may not be completely representative of the chronic metabolic alteration characteristic of liver cirrhosis. In experimental model of bile duct ligation (BDL), tumor necrosis factor‐*α* (TNF‐ *α*) has been suggested to play a potential role in the pathogenesis of muscle wasting mediating the activation of the ubiquitin‐proteasome pathway (Lin et al. 2005).

To the best of our knowledge, skeletal muscle mass modification is still not well characterized in experimental models of liver cirrhosis.

Therefore, the primary aim of this study was to characterize, at morphological and functional levels, skeletal muscle alterations secondary to liver cirrhosis induced in mice by either BDL or carbon tetrachloride (CCl4) administration. Secondarily, we identified possible molecular pathways involved in the promotion of skeletal muscle myopenia.

## Material and Methods

The experiments were performed in 12‐week‐old male wild‐type (FVB) mice purchased from Charles River Laboratories (Lecco, Italy). The mice were kept under constant temperature and humidity in a 12‐h controlled dark⁄light cycle. All the experiments were conducted within the animal welfare regulations and guidelines of the Italian national law D.L. 04/03/2014, n.26, about the use of animals for research.

### Mice models of chronic liver disease

#### Common bile duct ligation

The surgical procedure was performed under sterile conditions. Under tribromoethanol anesthesia, following a median abdominal incision, bile duct was exposed and then doubly ligated, once close to the liver hilus, once 2 cm below the liver hilus. The bile duct was then sectioned between these two ligatures. Mice were sacrificed at the end of 5 weeks of treatment by cervical dislocation. During this period, animals were fed ad libitum with standard food pellets (25/18 standard diet from Mucedola, Settimo, Milanese, Italy).

#### Administration of CCl_4_

Liver fibrosis was induced by intraperitoneal injection, twice a week, of CCl_4_ 0.2 mL/100 g in refined olive oil (1:1) for 12 weeks according to a well‐established model (Domenicali et al. 2009). Mice were sacrificed at the end of 12 weeks of treatment by cervical dislocation. During this period, animals were fed ad libitum with standard food pellets (25/18 standard diet from Mucedola, Settimo, Milanese, Italy).

#### Control mice

Mice undergoing median abdominal incision and exposure of the common bile duct without bile duct ligation represented the sham operated control for the BDL (CTR‐BDL). These animals were sacrificed after 5 weeks as the BDL.

Mice receiving intraperitoneal injections of vehicle only twice a week represented the control group for mice treated with CCl_4_ (CTR‐CCl_4_). These mice were sacrificed after 12 weeks.

At sacrifice, the presence of ascites was evaluated in all groups of animals and weighted using sterilized and already weighed paper disks. Ascites was then subtracted from the body weight to obtain the dry body weight.

### Histopathology of the liver

The liver was removed, weighed and immediately fixed in 10% phosphate‐buffered formalin and embedded in paraffin. Connective tissue was quantified by Sirius Red staining by analyzing liver sections with an Image Analysis System (IAS; Delta Sistemi, Rome Italy) and the morphological changes were measured by haematoxylin–eosin (H&E) staining. Intrahepatic bile duct mass (IBDM) was quantified by immunohistochemistry for cytokeratin‐19 (CK‐19) (Mancinelli et al. 2010).

### Skeletal muscle mass – histological and histochemical analysis

Immediately after animal sacrifice, the two gastrocnemius, quadriceps and tibialis muscles, were dissected and weighed. One muscle of each group was snap frozen in liquid nitrogen and stored at‐80°C, while the contralateral was used for the histological and histochemical analysis. Muscles were embedded in tissue freezing medium and snap frozen in nitrogen‐cooled isopentane. Sections of muscle tissue were cut at 7 microns in a −28°C cryostat and stained for hematoxylin and eosin (H&E). Morphometric examination was carried out with a microscope at 10× and 20× magnification, connected to a computerized image analysis system. Muscle cross‐sectional area of the quadriceps at its mid‐portion was calculated and total number of myofibers was counted. Boundaries of individual muscle fibers were delineated and fiber cross‐sectional area (CSA) was determined from the number of pixels within the outlined fiber using Image Analysis System (ImageJ 1.48). A minimum of four random fields (corresponding to about 1000 cross‐sectioned fibers) was photomicrographed for each quadriceps and mouse (>3 animals/groups).

### Ex‐vivo mice extensor digitorum longus mechanical measurements

Extensor digitorum longus (EDL) muscle isolated from the animal models above described was tested in vitro for its functional properties, both in isometric and isotonic conditions (Del Prete et al. 2008). Briefly, the muscle to be tested was transferred to a temperature‐controlled chamber (30°C), containing a Krebs‐Ringer solution equilibrated with 5% CO_2_‐95% O_2_. The proximal tendon was fixed to an actuator/transducer (model Aurora Scientific Instruments 300B).

The isolated muscle was electrically stimulated by means of two platinum electrodes with 300 mA controlled current pulses. Optimum muscle length was adjusted to the length (*L*_0_) that produced the highest twitch force. Single pulse stimulation was employed to evaluate the specimen's kinetic parameters, namely the maximum force derivative for the contraction (df/dt) and the relaxation (−df/dt) phases of the muscle contraction. Maximum tetanic force was measured with a train of pulses delivered at 180 Hz. Specific force was computed as maximum force divided by estimated muscle cross‐sectional area (Brooks and Faulkner 1988). Muscle resistance to fatigue was evaluated in isotonic conditions, allowing the specimen to shorten against a load equal to one‐third of its own maximum force.

### RNA extraction and quantitative RT‐PCR

Total RNA extraction from mice muscle tissue was performed with tissue lyser (Qiagen) in TriRiagentTM (Sigma). RNA was reverse‐transcribed using a QuantiTect Reverse Transcription kit (Qiagen). The reverse transcription reactions were performed according to the manufacturers' instructions. Quantitative PCR was performed on an ABI PRISM 7500 SDS (Applied Biosystems, USA), using pre‐made 6‐carboxyfluorescein (FAM)‐labeled TaqMan assays for GAPDH, MuRF‐1, Atrogin‐1, Myostatin, LC3 (Applied Biosystems). Quantitative RT‐PCR sample values were normalized to the expression of GAPDH mRNA. The relative level for each gene was calculated using the 2‐DDCt method (Livak and Schmittgen 2001) and reported as mean fold change in gene expression.

### Protein extraction

Protein extraction from tissue muscle of BDL, CCl_4_, and control mice was performed in Lysis Buffer (10 mmol/L Tris, 150 mmol/L Sodium Chloride, 1% NP40, 0.1% SDS, 10% Glycerol, 1% Deoxycholate, 1 mmol/L Phenylmethylsulfonyl Fluoride, 1 *μ*g/mL Aprotinin, 1 *μ*g/mL Leupeptin, 1 *μ*g/mL Pepstatin, 1 mmol/L Sodium Orthovanadate, and 1 mmol/L Sodium Fluoride). Equal amounts of protein from each lysate were separated in SDS polyacrilamide gel and transferred onto a nitrocellulose membrane. Filters were blotted over night (o.n.) with primary antibodies diluted in TBST, after 5% milk saturation. Then, filters were incubated with secondary antibodies anti‐Rabbit o anti‐Mouse (IgG HRP‐conjugated) (Bethyl, Montgomery, TX. Cat. A120‐201P) in 1% milk for 1 h. Signals were acquired by ChemiDoc MP instrument (Bio‐Rad, Hercules, CA) and densitometric analysis was performed with Image Lab acquisition analysis software (Bio‐Rad). The expression levels of each analyzed protein were calculated with respect to control group and reported as mean fold change values. Expression levels of the proteins of CTR‐BDL and CTR‐CCl_4_ mice were superimposable and for that reason, the two groups were considered together for the western blot analysis. Protein level of actin was used as control for equal protein loading. The following primary antibodies were used: Phospho‐Akt (Ser473) (D9E) XP (#4060) Cell Signaling; Akt Antibody (#9272), Cell Signaling; Phospho‐mTOR (Ser2448) (#2971), Cell Signaling; mTOR (#2972), Cell Signaling; Myostatin (sc‐6885‐R), Santa Cruz; Actin (A2066), Sigma Aldrich; LC3B (#2775), Cell Signaling; NF‐κB p65 (C22B4) (#4764), Cell Signaling; Phospho‐NF‐κB p65 (Ser536) (7F1) (#3036), Cell Signaling; MuRF1 (C‐terminal region) (MP3401), ECM Biosciences; Anti‐SQSTM1 / p62 (ab56416), Abcam.

### Statistical analysis

All data are expressed as mean ± SEM. Statistical analysis was performed with GraphPad Prism v 6.05 software; Student's unpaired *t*‐test for parametric data and Mann–Whitney–Wilcoxon test for nonparametric data were used when two groups were compared. The difference between the two groups was considered significant for *P *<* *0.05. Mann–Whitney Rank Sum test was used to compare fiber size frequency distributions. The difference in the median values between the two groups was considered significant for *P* < 0.05.

## Results

### Liver histology and muscles morphological analysis in experimental groups

**Figure 1:**
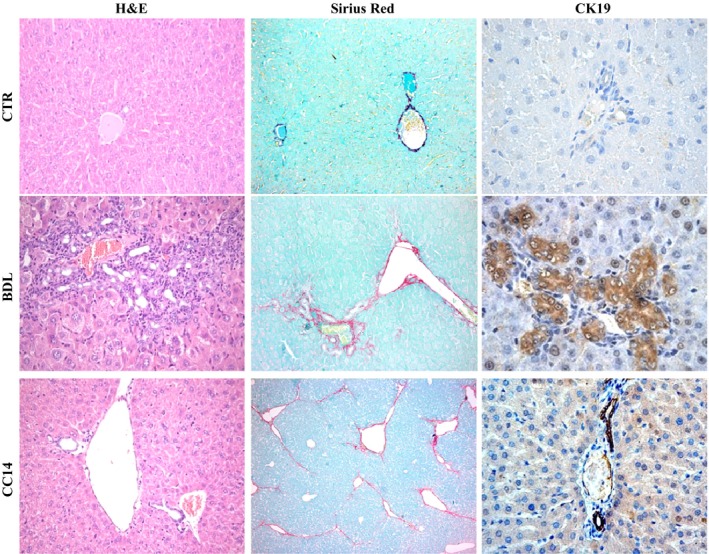
Liver histology in experimental groups. Histology of liver sections showed enhanced fibrosis (evaluated by Sirius Red Staining) and an increased intrahepatic bile duct mass (BDM) expressed by positive cytokeratin 19 in BDL and CCl_4_ mice with respect to their own controls. CTR, control; BDL, bile duct ligation; CCl_4_, Carbon Tetrachloride.

In this study, the mice typically developed liver cirrhosis/ductular proliferation 12 weeks after CCl_4_ injection and 5 weeks after BDL, respectively, which was confirmed by histological analysis (Table 1 and Fig. 1). No liver damage or histological alteration was identified in CTR mice (Fig. 1).

**Table 1:** Evaluation of liver fibrosis and bile duct mass in experimental groups

	Volume fraction occupied by collagen (sirius red stained) fibers	BMD
CTR	1.9 ± 0.21	0.28 ± 0.05
BDL	5.4 ± 1.1^a^	4.18 ± 0.2^a^
CCl_4_	7.8 ± 0.5^a^	0.21 ± 0.08

Values are mean ± SEM.

BMD, bile duct mass; CTR, control; BDL, bile duct ligation; CCl_4_, Carbon Tetrachloride. *n* ≥ 3.

a *P* < 0.05 vs. CTR.

All CCl_4_ animals developed ascites at the end of the treatment, while no ascites was present in the BDL model. At sacrifice, ascites was estimated to be 0.135 ± 0.020 mg.

**Figure 2:**
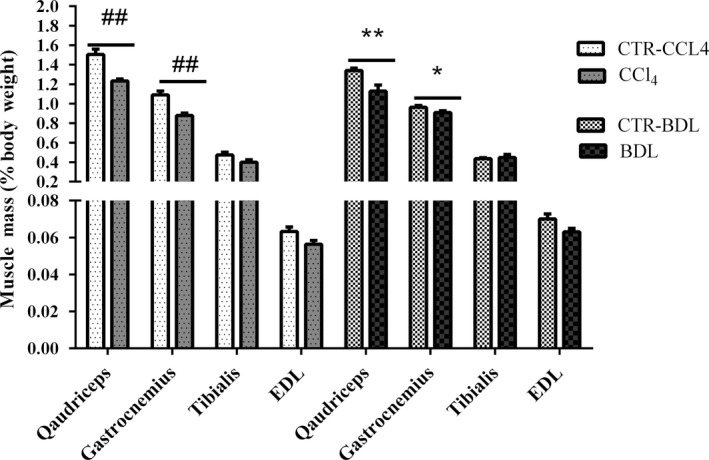
Muscles weight in experimental groups. Measurements are presented as mean ± SEM (^##^ *P* < 0.01 vs CTL‐CCl4; *P < 0.05 and **P < 0.01 vs CTL‐BDL).

The weight of total body, liver, spleen, lower extremities muscle, as well as the organ‐to‐body weight ratios are shown in Table 2. The initial body weights of CCl_4_ and BDL animals were similar with respect to their controls but at the end of the study, CCl_4_ animals tended to weigh more than CTL‐CCl_4_ ones, while BDL tended to weigh less than their controls even if these modifications did not reach statistical significance (*P* = 0.24 and *P* = 0.05, respectively). The liver and spleen‐body weight ratios were significantly higher both in CCl_4_ animals and BDL with respect to their controls. Muscle‐to‐body weight ratio of the total lower extremities muscles was significantly lower both in the CCl_4_ and BDL animals compared with their controls. A significant reduction in muscles weight was observed in gastrocnemius and quadriceps muscles (Fig. 2).

**Table 2:** Organ and muscle weights in experimental groups

	CTR‐CCl_4_	CCl_4_	*P*	CTR‐BDL	BDL	*P*
Preoperative weight, g	27.96 ± 0.63	26.61 ± 0.60	0.18	29.67 ± 0.67	29.26 ± 0.41	0.79
Postoperative weight, g	29.04 ± 1.15	31.26 ± 0.91	0.24	30.48 ± 0.78	27.53 ± 1.20	0.05
Liver weight, mg	1.29 ± 0.11	1.72 ± 0.12	0.01^a^	1.34 ± 0.06	2.42 ± 0.23	0.0008^a^
Liver‐to‐body weight ratio	4.43 ± 0.23	5.60 ± 0.30	0.01^a^	4.41 ± 0.18	8.72 ± 0.53	0.0002^a^
Spleen weight, mg	0.144 ± 0.02	0.148 ± 0.005	0.49	0.154 ± 0.01	0.268 ± 0.02	0.0007^a^
Spleen‐to‐body weight ratio	0.417 ± 0.01	0.479 ± 0.03	0.04^a^	0.441 ± 0.02	0.968 ± 0.05	0.0001^a^
Lower extremity muscles weight, mg	923.2 ± 39.2	809.4 ± 10.2	0.04^a^	866.5 ± 17.5	711.17 ± 30.6	0.0033^a^
Lower extremity muscles weight‐to‐body weight ratio	3.13 ± 0.11	2.59 ± 0.06	0.002^a^	2.85 ± 0.039	2.59 ± 0.09	0.031^a^

Values are expressed as mean ± SEM.

CTR, control; BDL, bile duct ligation; CCl_4_, Carbon Tetrachloride. *n* = 7.

a *P* < 0.05 versus CTR.

**Figure 3:**
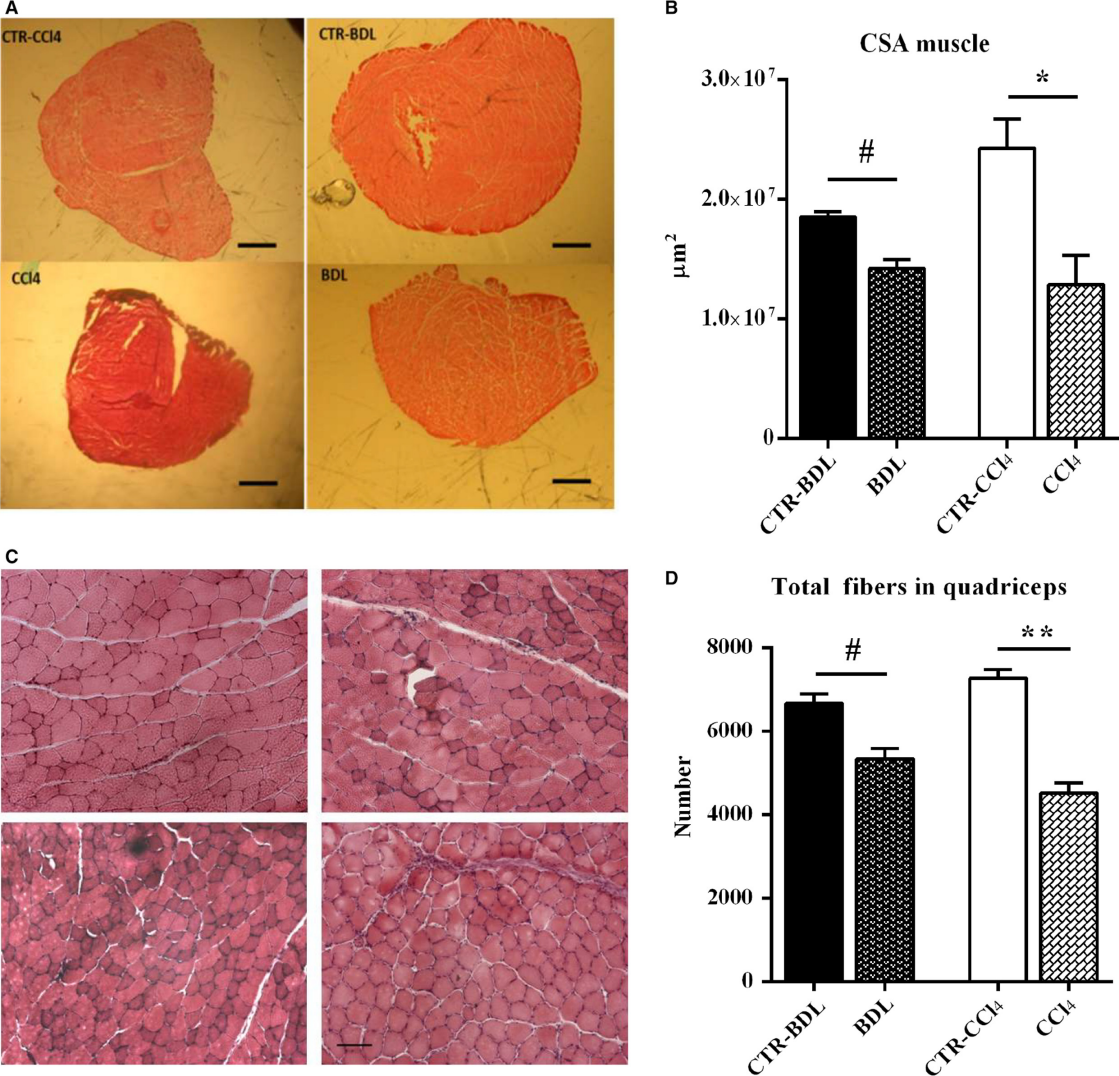
Histological analysis of quadriceps from the experimental groups. (A) Haematoxylin and eosin‐stained cross‐sectional area of quadriceps muscles at its mid‐portion from the experimental groups. Bar = 10 *μ*m. (B) Median values of quadriceps cross‐sectional area from the experimental groups (*n* ≥ 3); (C) Haematoxylin and eosin‐stained cross‐sections of myofiber of quadriceps muscles from the experimental groups. Bar = 100 μm.; (D) The myofiber number of the quadriceps muscles from the experimental groups (*n* ≥ 3); (E–F) Fiber size of quadriceps muscles in CTR‐CCl_4_ (*n* ≥ 4) and CCl_4_ (*n* ≥ 4). Median values of fiber size. (Mann–Whitney Rank Sum test: *P* < 0.0001). Measurements are presented as mean ± SEM (^#^ *P* < 0.05 and ^##^ *P* < 0.01 vs. CTL‐CCl4; *P < 0.05 and **P < 0.01 vs. CTL‐BDL). CTR, control; BDL, bile duct ligation; CCl_4_, Carbon Tetrachloride.

**Figure 4:**
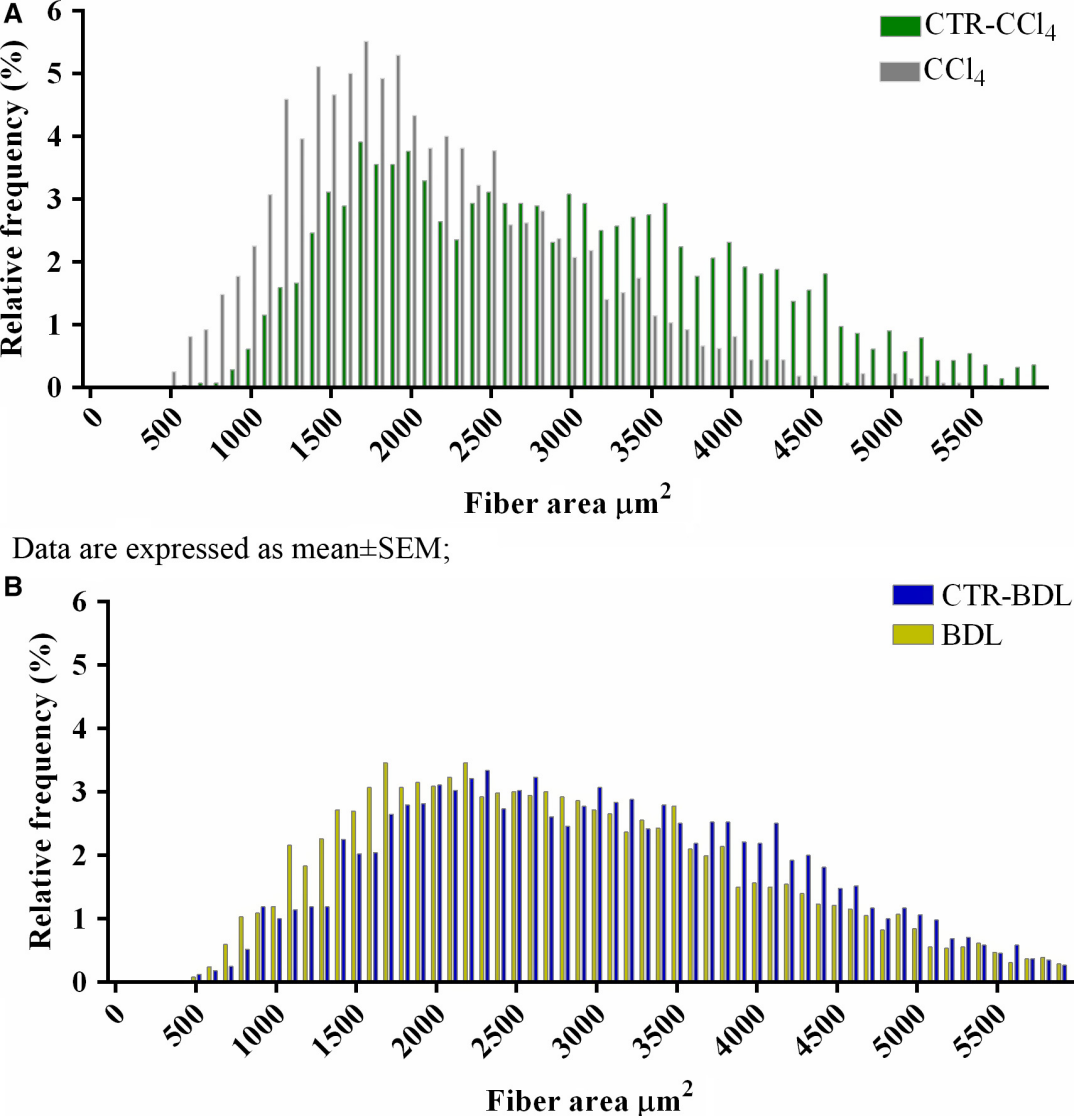
Frequency distribution of muscle fibers' cross‐sectional area of quadriceps from the experimental groups. A: CTR‐CCl_4_ vs CCl_4_; CTR‐CCl_4_: 2903 ± 22; CCl_4_: 2116 ± 17, *P* < 0.0001; B: CTR‐BDL vs BDL; CTR‐BDL: 2990 ± 17, BDL: 2774 ± 17, *P* < 0.0001. Measurements are presented as mean ± SEM Abbreviations: CTR: control; BDL: bile duct ligation; CCl4: carbon tetrachloride.

To verify whether or not skeletal muscle myopenia occurred in our experimental models, we carried out histological analysis of skeletal muscles (Fig. 3). The average cross‐sectional area (CSA) of the quadriceps was significantly reduced with respect to controls both in CCl_4_ (−47% vs. CTR‐ CCl_4_) and in BDL (−34% vs. CTR‐BDL) (Fig. 3A–B). The total myofiber number of quadriceps of cirrhotic mice was also lower compared to controls (Fig. 3C–D). The frequency distribution of muscle fibers' CSA revealed a shift toward a smaller size of muscle fibers in mice treated with CCl_4_ and BDL, which was more evident for CCl_4_ (Fig. 4). The results suggested the development of a condition of skeletal muscle myopenia in cirrhotic mice.

### Functional performance and contractile properties of skeletal muscles in experimental groups: ex‐vivo analysis

To determine whether liver cirrhosis affected the capacity to produce force and the contractile properties of skeletal muscles, a study of mechanical parameters was performed on the EDL, a typical fast‐type fiber muscle, of both BDL and CCl_4_ mice and of their controls.

**Figure 5:**
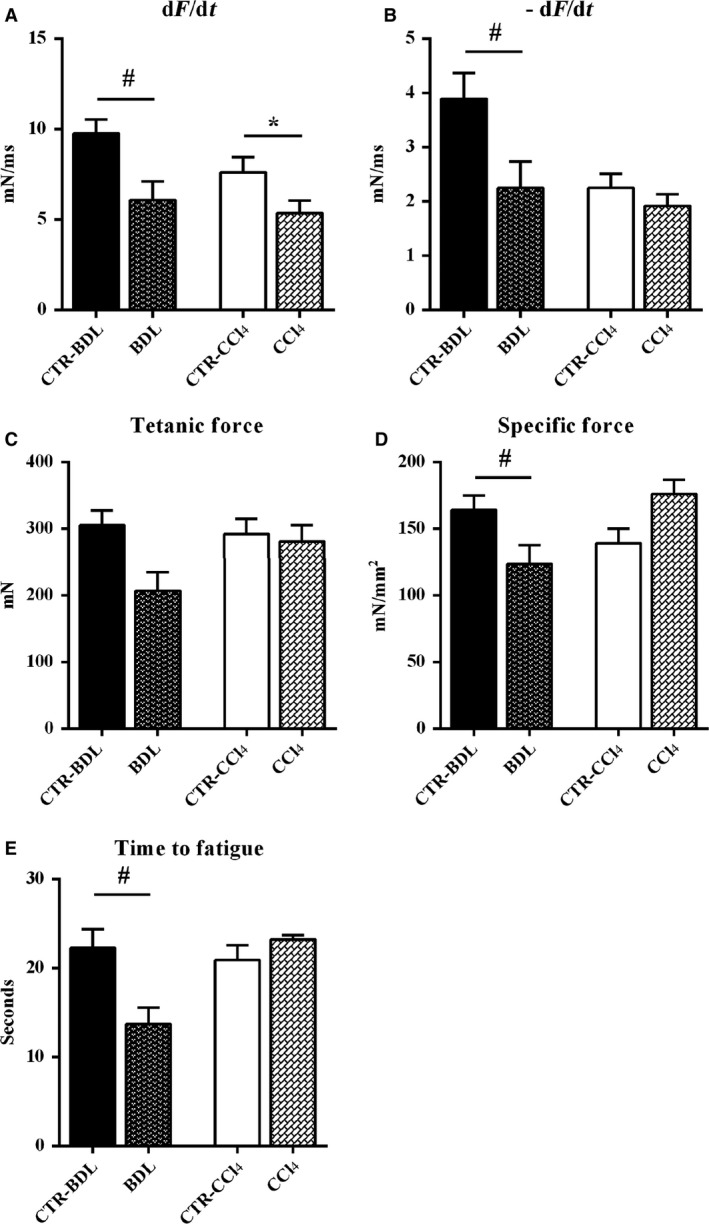
Physiological properties of extensor digitorum longus muscles from experimental groups. Functional performance and contractile properties of extensor digitorum longus muscles were compared by dF/dt, −dF/dt, tetanic force, specific force, and time to fatigue. All measurements are presented as mean ± SEM (^#^ *P* < 0.05 vs. CTR‐CCl_4_; *P < 0.05 vs. CTR‐BDL); *n* ≥ 3; BDL, bile duct ligation; CCl_4_, Carbon tetrachloride.

Among the functional parameters analyzed, we observed a slowing down of the kinetic properties of EDL muscle, as expressed by the dF/dt (Fig. 5A) and the −dF/dt (Fig. 5B). Of note, the BDL treatments induced more severe functional alterations, compared with CCl_4_ treatments, as revealed by the significant reduction in the tetanic force, the reduction in specific force, and by the significant reduction in the time to fatigue (−38%) (Fig. 5C–E). No direct significant effects of CCl_4_ treatment seems to appear in the specific force.

### Characterization of the main molecular pathways involved in liver cirrhosis‐induced myopathy

**Figure 6:**
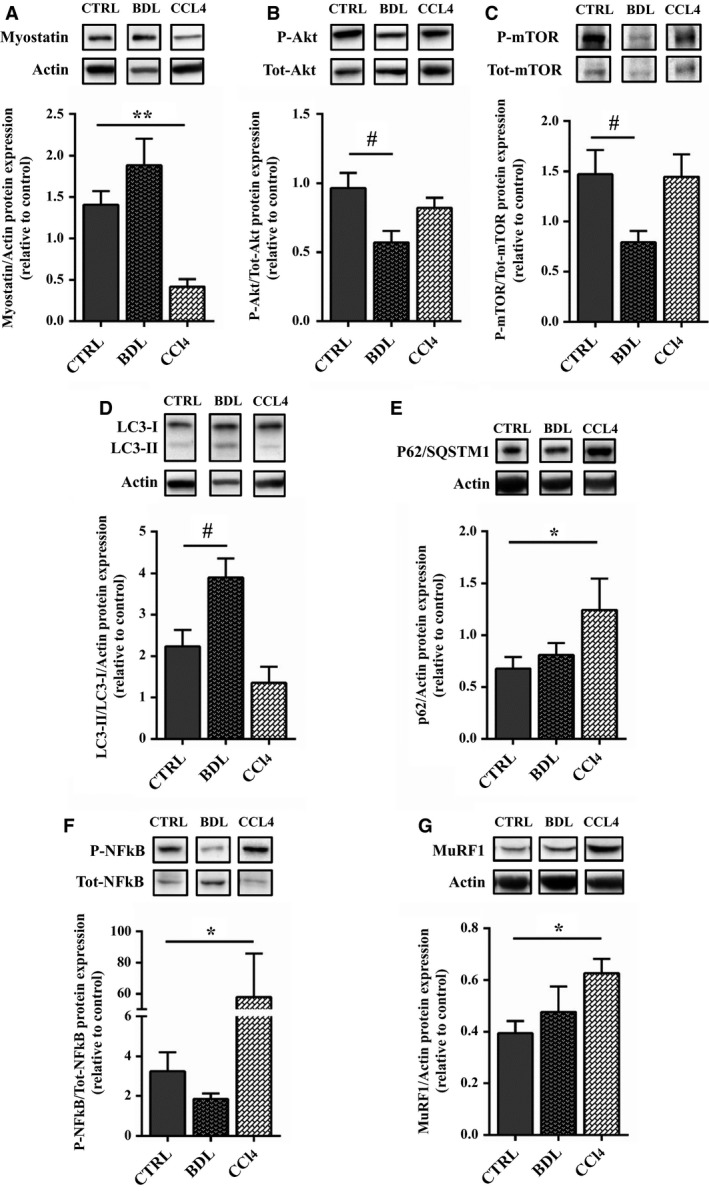
Representative western blot analysis in the experimental groups. Representative western blot analysis for (A) Myostatin and Actin expression (upper panel) and (lower panel) densitometric analysis of the ratio (Myostatin/Actin expression); (B) phospho‐Akt (Ser 473) and total Akt expression (upper panel) and densitometric analysis of the ratio between the phosphorylated and the total protein (lower panel); (C) phospho‐mTOR (Ser2448) and total mTOR expression (upper panel) and densitometric analysis of the ratio between the phosphorylated and the total protein (lower panel); (D) LC3 and Actin expression (upper panel) densitometric analysis of the ratio of LC3‐II/LC3‐I normalized to the expression of Actin; (E) p62/SQSTM1 and Actin expression (upper panel) and (lower panel) densitometric analysis of the ratio (p62/Actin expression); (H) phospho‐NF‐kB (Ser536) and total NF‐kB expression (upper panel) and densitometric analysis of the ratio between the phosphorylated and the total protein (lower panel); (I) MuRF1 and Actin expression (upper panel) and (lower panel) densitometric analysis of the ratio (MuRF1/Actin expression). The representative bands come from noncontiguous lanes. Data are represented as mean ± SEM; *n* = 6–8 per group. **P < 0.005; *P < 0.05 between BDL and control group; ^##^ *P* < 0.005; ^#^ *P* < 0.05 between CCl_4_ and control group (by Mann–Whitney test). BDL, bile duct ligation; CCl_4_, Carbon Tetrachloride.

Myostatin, a member of TGF‐β superfamily, has been identified to play a pivotal role in mediating muscle atrophy in experimental models of liver damage. Western blot analysis revealed over expression of myostatin in BDL versus CTR while it was significantly reduced in CCl_4_ mice versus CTR (Fig. 6A). Myostatin negatively regulates the activity of the Akt‐mTOR axis, which promotes protein synthesis. In our models, a significant decrease in Akt expression and mTOR was observed in BDL mice (Fig. 6B–C), while no modification was observed in CCl_4_‐treated mice.

**Figure 7:**
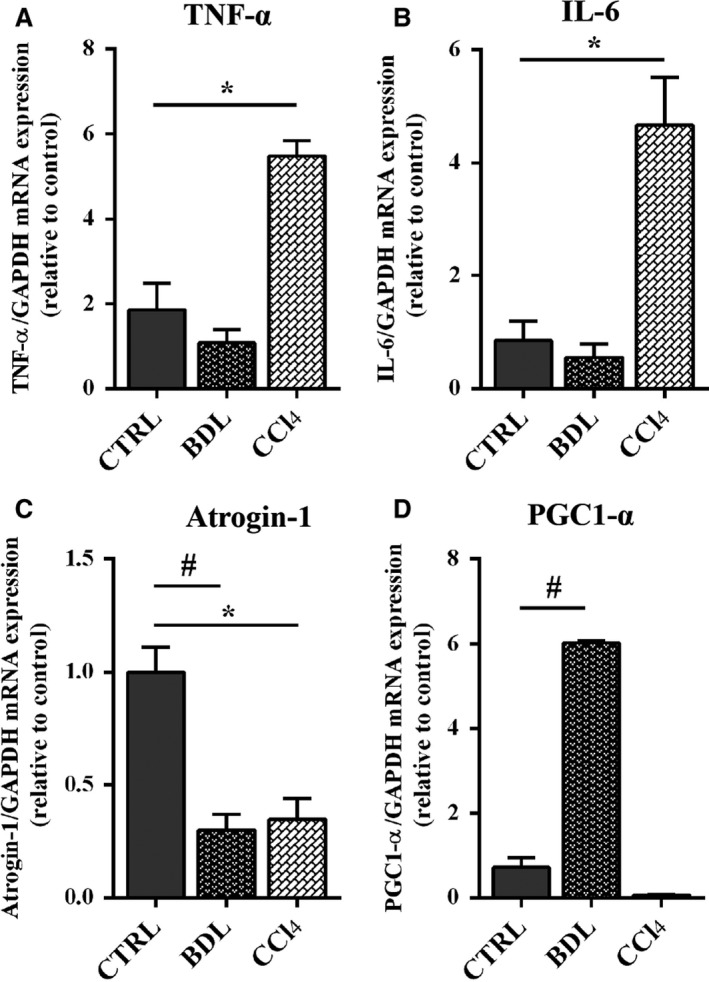
Expression of myogenic genes in experimental groups. Real‐time PCR analysis for the expression of TNF *α* (F), IL‐6 (G) Atrogin‐1 (L), and PGC1‐ *α* (M) on tissue muscle of BDL, CCL4, and control mice. Data are represented as mean ± SEM; *n* = 3–6 per group. **P < 0.005; *P < 0.05 between BDL and control group; ^##^ *P* < 0.005; ^#^ *P* < 0.05 between CCl_4_ and control group (*by* Mann*–*Whitney test*)*. BDL, bile duct ligation; CCl_4_, Carbon Tetrachloride.

Autophagy is responsible for controlled degradation of cytoplasmic components, as well as providing essential nutrients during starvation and stress to maintain cellular homeostasis. Since cirrhosis is a state of accelerated starvation, enhanced muscle autophagy may serve as a source of essential amino acids for critical cellular function. Key components of this pathway include a cytosolic protein (LC3‐I), which is also modulated by myostatin expression, that is lapidated (LC3‐II) to facilitate autophagosome membrane formation, and p62/SQSTM1 that targets cargo to the autophagosome for degradation. LC3 expression was increased in BDL mice and reduced in CCl4 mice (Fig. 6D), while p62/SQSTM1 protein's levels were overexpressed in CCl_4_ mice but not in BDL (Fig. 6E). These data suggested that other pathway, myostatin‐independent, able to activate proteolytic mechanisms were involved in muscle myopathy in CCl_4_ mice. Expressions of inflammatory cytokines such as TNF‐*α* and IL‐6 were evaluated. TNF‐*α* has been shown to have direct catabolic effect on skeletal muscle and produces muscle atrophy through the upregulation of the transcription factor NF‐κB which activates transcription of components of the ubiquitin–proteasome proteolytic pathway such as atrogin‐1 and MuRF‐1. A significant increase in TNF‐*α* and IL‐6 was observed in CCl_4_ mice, while no modification was observed in BDL mice (Fig. 7A–B). Accordingly pNF‐kB expression, the activated form of NF‐kB, was increased in CCl_4_ mice (Fig. 6F). MuRF‐1 expression was also confirmed to be increased in CCl_4_ mice, while it was unchanged in BDL models (Fig. 6G). Real‐time PCR showed reduction in Atrogin‐1 transcription both in CCl_4_ and BDL (Fig. 7C). We thus speculated that atrogin‐1 expression can be partially repressed and other pathways may be involved in the CCl_4_‐ and BDL‐mediated muscle atrophy. The first hypothesis is supported by the evidence that PGC1 *α*, which increased in BDL muscles (Fig. 7D), inhibits FoxO‐dependent transcription on atrogin1 promoter, thereby suppressing atrogin1 expression (Sandri et al. 2006).

## Discussion

With this study, we have characterized skeletal muscle alterations secondary to liver cirrhosis in two different experimental models. Common BDL is a model of secondary biliary cirrhosis, and although it has been mainly employed in rats (Alvaro et al. 2000; Mancinelli et al. 2010), it has also been developed in mice (Chang et al. 2005). As demonstrated by histological analysis performed on liver sections, marked cholangiolar proliferation and expansive portal fibrosis was observed 5 weeks after BDL in all the mice included in the study. In a recent paper by Domenicali and colleagues, CCl_4_ administration in mice has been proven to be a successful model to establish a frank cirrhosis ensuring optimal results in terms of reproducibility, tolerability, and time of induction (Domenicali et al. 2009). Indeed, after treatment, all the animals included in this study showed a fully established cirrhosis without significant differences in the amount of liver fibrosis and all of them developed ascites. Both BDL and CCl_4_‐induced cirrhosis were characterized by modifications in muscle mass. The decrease ratio of muscle weight to body weight together with the histological evidence of a reduction in the muscle CSA and muscle fibers associated with a shift of fibers' CSA toward smaller size of muscle fibers are all evidences of the establishment of a condition of myopenia. We therefore suggest that both models can be employed to explore mechanism of skeletal muscle myopenia secondary to liver cirrhosis.

It is well established that many patients with cirrhosis experience decreased strength and increased fatigue of skeletal muscle (Jones et al. 2012). This is mostly seen as part of a poor nutritional status, enhanced catabolism, or decreased protein synthesis, but specific investigations of muscle function as well as of muscle composition in liver cirrhosis are still lacking. Fatigue and muscle weakness can depend on central or peripheral process: the clinical expression of fatigue and muscle strength encompasses complex interactions with biological, psychosocial, and behavioral processes (Allen et al. 2008). For these reasons, in human study, it is extremely difficult to differentiate at which points weakness arises. Experimental models and, most of all, the availability of isolated muscle tissue offer the opportunity to investigate pathological mechanism responsible for muscle fatigue and weakness directly. Data actually available on this argument in models of liver cirrhosis are still insufficient. Previous experimental studies have described nonspecific histology changes in skeletal muscle, most often myocytolysis, with variation in fiber size and type II fiber atrophy (Gayan‐Ramırez et al. 1998). However, most of the studies were mainly related to alcohol myopathy that is probably characterized by specific alterations. In contrast, the association between myopathy and nonalcoholic liver cirrhosis is less well documented (Gayan‐Ramırez et al. 1998; López‐Lirola et al. 2003; Weber et al. 1992), and the possible pathogenesis is poorly understood. In a study by Ramirez and colleagues, diaphragm and peripheral muscle mass in BDL cirrhotic rats were decreased and a clear reduction in the cross‐sectional area of type IIx/b muscle fiber was also present in both diaphragm and gastrocnemius. However, no changes in diaphragm force nor in its endurance were observed (Gayan‐Ramırez et al. 1998). Type IIb fiber atrophy was also reported by Weber et al. (1992) in diaphragm sections of rat model in which liver damage was induced by CCl4 administration. Similar results were achieved by López‐Lirola et al. (2003)who found that muscle atrophy ensues in carbon tetrachloride‐treated rats, especially IIa fiber atrophy, but only when protein deficiency was also present, suggesting that fiber atrophy was predominantly as a consequence of malnutrition but not of cirrhosis itself. In all these studies, functional analysis was not performed. In our study, the evaluation of the functional performance and the contractile properties of skeletal muscles, performed on EDL, revealed significant alterations of muscle functional parameters in both models of liver damage. BDL compromised both muscle force and muscle contraction velocity, while cirrhosis secondary to CCl_4_ administration compromised the contraction velocity without affecting muscle force. These data, suggest that BDL and CCl_4_ secondary cirrhosis are both associated with the development of skeletal muscle myopenia but the functional characteristics of muscle impairment is different in the two models.

The pathogenesis of muscle myopenia has also been investigated. Previous studies on experimental models of hyperammonemia or portocaval shunt in rats have demonstrated that the predominant mechanism of muscle depletion in these disorders were the increased activation of NF‐kB p65, with a subsequent higher expression of myostatin, a negative regulator of skeletal muscle mass (Qiu et al. 2013).

Moreover, muscle wasting was associated with activation of autophagic pathways, while proteolytic pathways were found to be involved in the early phase of liver damage in association with a reduction in the expression of IGF‐1, a positive regulator of muscle mass (Dasarathy et al. 2004, 2011, 2007).

**Figure 8:**
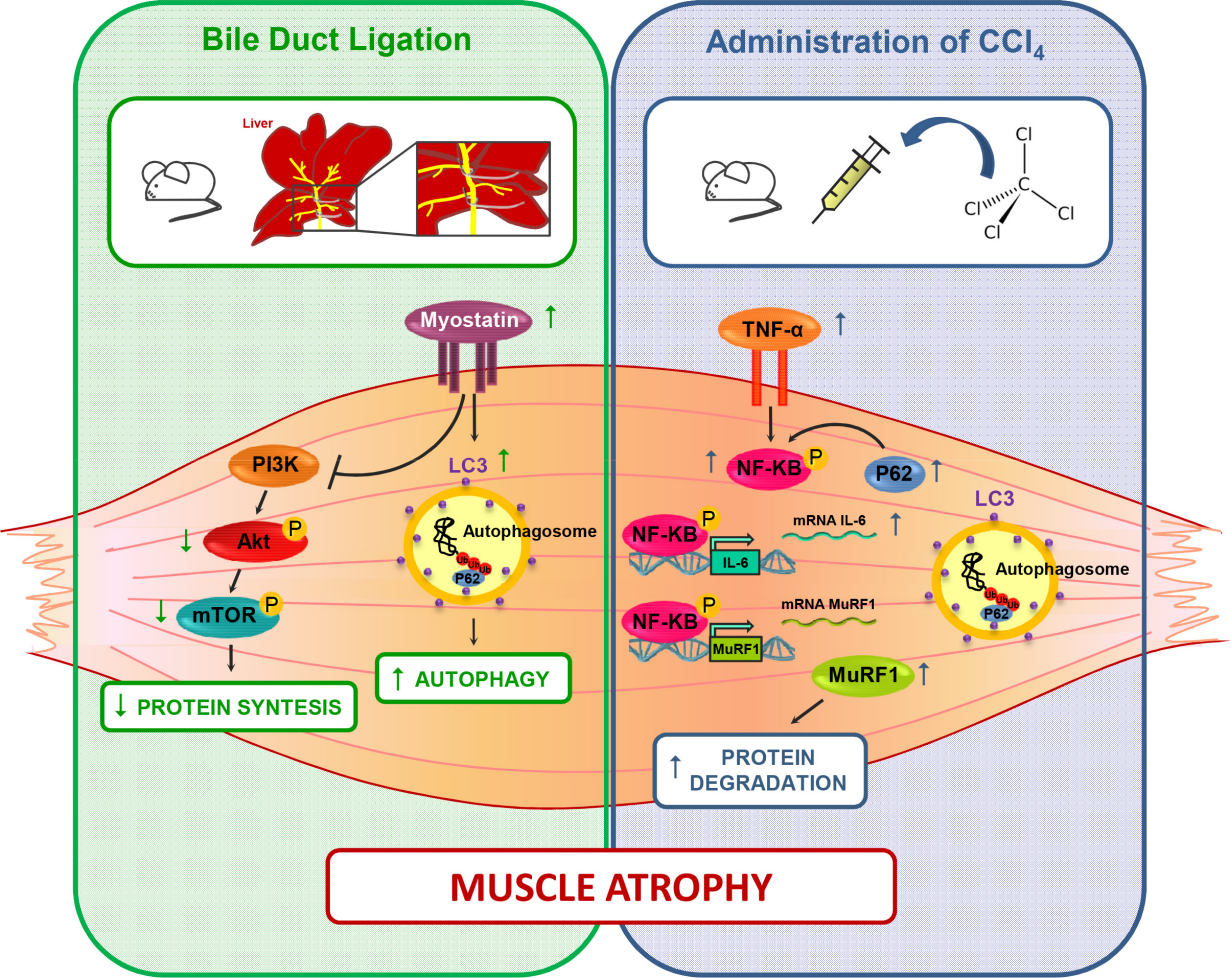
Molecular pathways in experimental groups. A summary of the molecular pathways responsible for skeletal muscle atrophy in the different experimental models of cirrhosis are described here. BDL, bile duct ligation; CCl_4_, Carbon Tetrachloride.

However, no evidence of the pathological mechanism responsible for muscle mass modifications in other models is actually available. In our study, we tried to evaluate potential pathways involved in skeletal muscle myopathy. The reduction in the muscle cross‐sectional area of treated mice would be expected to activate relevant gene involved in the induction of muscle atrophy such as MAFbx/Atrogin‐1 and MuRF‐1 (Sandri et al. 2004). Surprisingly, quantitative reverse transcription polymerase chain reaction (RT‐PCR) analysis revealed that while MuRF‐1 expression increased in CCl_4_ and BDL compared to CTR mice, atrogin‐1 was downregulated. We extended the study by analyzing several other factors that can modulate these factors and therefore muscle atrophy. We thus speculated that Atrogin‐1 expression can be partially repressed and/or other pathways may be involved in the CCl_4_‐and BDL‐mediated muscle atrophy. The first hypothesis is supported by the evidence that PGC1 *α*, which increased in BDL muscles, inhibits FoxO‐dependent transcription on Atrogin‐1 promoter, thereby suppressing atrogin‐1 expression (Sandri et al. 2006). We also defined the critical and specific mediators and signaling activated by the two distinct treatments, namely CCl_4_ and BDL that might trigger muscle atrophy. Interestingly, we demonstrated that BDL activates myostatin, a negative modulator of muscle mass, which also negatively regulates the activity of the Akt pathway (Rodriguez et al. 2014). Indeed, we also demonstrated a selective down modulation of the Akt, a kinase essential to promote protein synthesis and cell survival and to block protein degradation, and of the mTOR, which is an important mediator for the positive control of protein synthesis and the maintenance of the differentiated myogenic phenotype. In addition, we observed an upregulation of LC3, a molecular marker of autophagic pathway, which is also modulated by myostatin expression (Rodriguez et al. 2014). The lack of p62 upregulation suggests that BDL induce an accumulation of autophagosomes, which however do not fuse with lysosomes. Conversely, CCl_4_ activates alternative pathways. We observed a selective upregulation of TNF‐a and IL‐6, two cytokines which activate NF‐kB, a signaling pathway linked to several pathologic processes in skeletal muscle (Bar‐Shai et al. 1057). NF‐kB, in turn maintains upregulated IL‐6, contributing to activate the inflammation amplifier and therefore exacerbating the atrophic phenotype. Of note, we also analyzed NF‐kB based on the interesting evidence that it induces muscle atrophy and wasting, upregulating MuRF‐1 but not Atrogin‐1 (Cai et al. 2004; Mourkioti et al. 2006). Interestingly, it has been also reported that p62, which we observed upregulated in CCl4 muscle, can promote the expression of inflammatory genes via NF‐kB (Moscat and Diaz‐Meco 2009). The main molecular pathways responsible for skeletal muscle myopenia in the two different experimental models of cirrhosis are summarized in Figure 8. The main shortcoming of our study was the lack of a pair‐fed controls for both BDL and CCl_4_ mice; nevertheless, all the animals received a similar diet, so the effect of malnutrition/protein deficiency per se could not be assessed. Moreover the inclusion of time‐course experiments and of gender as additional key parameter would have added information on the dynamic process leading to skeletal muscle myopenia and on the different feature of muscle wasting among gender usually observed in clinical study.

In conclusion, in our study, we observed skeletal muscle myopenia in both the experimental models, reduction in protein synthesis and activation of protein degradation are the main mechanisms responsible for myopenia in BDL mice, while activation of ubiquitin‐pathway through inflammatory cytokines seems to be the main potential mechanism involved in CCl_4_ mice.

## Conflict of Interest

The authors have nothing to disclose.
